# Effect of Hafnium-Based Thin Film Thickness on Microstructure and Electrical of Yttrium-Doped Hafnium Oxide Ferroelectric Devices Prepared by Magnetron Sputtering

**DOI:** 10.3390/mi16091066

**Published:** 2025-09-21

**Authors:** Bei Ma, Ke Ma, Xinhui Qin, Yingxue Xi, Jin Zhang, Xinyu Yang, Pengfei Yang, Weiguo Liu

**Affiliations:** 1 School of Optoelectronic Engineering, Xi’an Technological University, Xi’an 710021, Chinamk828136@163.com (K.M.); j.zhang@xatu.edu.cn (J.Z.); pfyang@xatu.edu.cn (P.Y.);; 2 Xi’an Plasma Advanced Optical Manufacturing International Science and Technology Cooperation Base, Xi’an 710021, China

**Keywords:** magnetron sputtering, hafnium oxide-based thin films, yttrium doping, thickness, microstructure, electrical properties

## Abstract

This study employs reactive magnetron sputtering technology to fabricate TiN/Y-HfO_2_/TiN multilayer thin film devices using titanium targets and yttrium-doped high-purity hafnium targets. A systematic investigation was conducted to explore the influence of hafnium-based thin film thickness on the structural and electrical properties of TiN/Y-HfO_2_/TiN thin film devices. Radio frequency magnetron sputtering was utilized to deposit Y-HfO_2_ films of varying thicknesses on TiN electrodes by controlling deposition time, with a yttrium doping concentration of 8.24 mol.%. The surface morphology and crystal structure of the thin films were characterized using atomic force microscopy (AFM), Raman spectroscopy, X-ray diffraction (XRD). Results indicate that as film thickness increases, surface roughness and Raman peak intensity increase correspondingly, with the tetragonal phase (t) characteristic peak being most prominent at 65 nm. DC magnetron sputtering was employed to deposit TiN top electrodes, resulting in TiN/Y-HfO_2_/TiN thin film devices. Following rapid thermal annealing at 700 °C, electrical properties were evaluated using a ferroelectric tester. Leakage current density exhibited a decreasing trend with increasing film thickness, while the maximum polarization intensity gradually increased, reaching a maximum of 11.5 μC/cm^2^ at 120 nm.

## 1. Introduction

With the rapid development of technologies such as 5G, big data, and the Internet of Things (IoT), there is an urgent market demand for new semiconductor devices that combine low power consumption, high-speed read/write capabilities, and high reliability. This is crucial for achieving high-performance computing. Ferroelectric materials, with their spontaneous polarization and polarization reversal properties, show great potential in non-volatile storage and logic operations [1]. As an emerging ferroelectric system, hafnium oxide (HfO_2_) thin films have garnered significant attention for effectively overcoming the compatibility issues between traditional perovskite ferroelectric materials and CMOS processes, as well as the degradation of ferroelectric properties at the nanoscale [2,3]. Recent studies have shown that by controlling element doping (such as Zr, Y) and annealing processes, ferroelectricity can be induced under specific conditions. This discovery has injected new vitality into ferroelectric material research and opened up new avenues for developing high-performance electronic devices [4]. To better understand the electrical properties of ferroelectric HfO_2_-based devices, it is necessary to reveal their fundamental mechanisms at the microscopic physical level. At the atomic scale, the formation of ferroelectricity in HfO_2_ thin films (primarily originating from the metastable orthorhombic phase) is accompanied by significant crystal phase transitions. The introduction of dopant atoms (such as Zr) and annealing processes induce specific lattice distortions by altering local bonding configurations and atomic coordination environments, and these distortions are key to the generation of ferroelectricity [5]. In Zr-doped HfO_2_, the slight difference in ion radius between Zr^4+^ and Hf^4+^ introduces local stress into the lattice, driving the crystal structure to transition from a non-ferroelectric phase to a ferroelectric phase [6].

Ekaterina Yurchuk et al. [7] investigated the ferroelectricity of Si-doped HfO_2_ thin films in relation to film thickness, Si concentration, and annealing temperature. The study found that for 9 nm-thick films, reducing Si content and increasing annealing temperature improves residual polarization; however, when the film thickness increases to 27 nm, residual polarization significantly decreases. This change in ferroelectric properties with thickness primarily stems from the differing crystallization behavior between thick and thin films [8]. Jaszewski et al. [9] explored the stability of the ferroelectric phase from the perspective of grain size. Their theoretical and experimental results indicate that a specific grain size range is favorable for stabilizing the metastable orthorhombic ferroelectric phase (o-phase). As the film thickness increases, the fraction of the non-ferroelectric monoclinic phase (m-phase) rises, and the phase composition can be optimized by controlling the deposition process.

Mimura et al. [10] used pulsed laser deposition (PLD) technology to prepare 15 nm thick Hf_0.93_Y_0.07_O_2_ thin films on (111) ITO/(111) YSZ single crystal substrates [11]. The results indicated that when the annealing temperature was below 600 °C, the film retained the monoclinic phase (m phase); when heat-treated in the 600–950 °C range, part of the m phase transforms into the tetragonal phase (t phase), but during subsequent cooling, the film transforms into a mixture of the monoclinic phase (m phase) and the orthorhombic phase (oIII phase) [11]. When the heat treatment temperature exceeds 950 °C, the initial m phase completely transforms into the t phase and ultimately stabilizes as the oIII phase after cooling [11]. These results indicate that by controlling the kinetic pathways of the heat treatment process, the transformation of the t phase into the oIII phase can be effectively promoted, thereby facilitating the attainment of a more desirable oIII phase in HfO_2_-based thin films [11].

Magnetron sputtering has become one of the primary methods for preparing HfO_2_ ferroelectric thin films. In 2012, Olsen et al. successfully prepared Y-doped HfO_2_ ferroelectric thin films using co-sputtering physical vapor deposition [12]. This method effectively avoids the influence of ALD experimental parameters on the ferroelectric properties of the film, and the films prepared by magnetron sputtering exhibit high density, stable composition, and low contaminant content [13].

## 2. Materials and Methods

**Figure 1:**
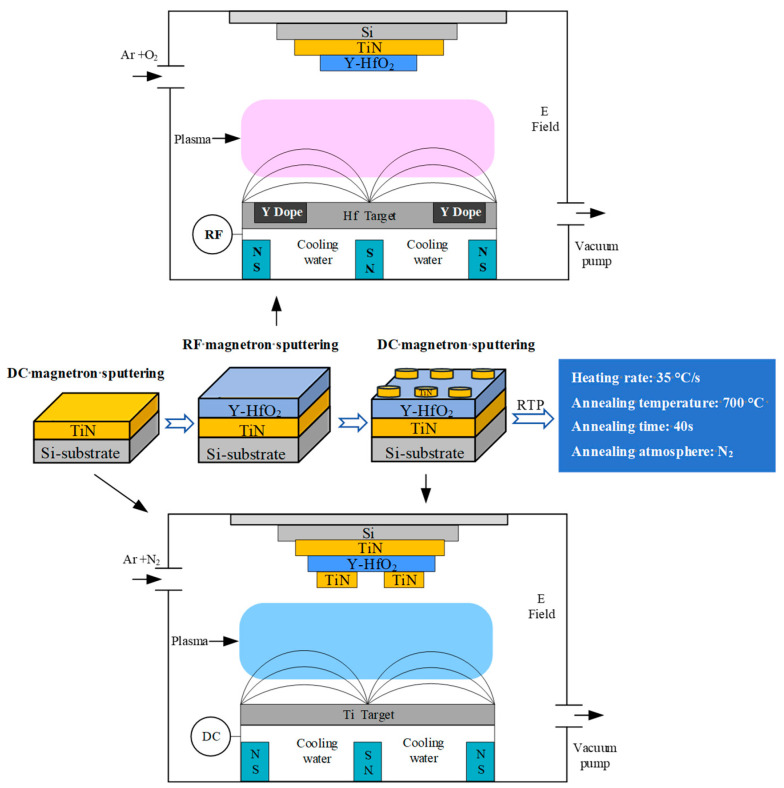
Process flow diagram for the preparation of the MFM (TiN/Y-HfO_2_/TiN/Si) capacitor structure.

The thin film deposition equipment used in this study is the MSP-400B high-vacuum magnetron sputtering coating equipment manufactured by Beijing Chuangshi Micro-Nano Technology Co, Ltd. (Fengtai District, Beijing, 100070, China). The preparation process is shown in Figure 1. First, a complete TiN thin film electrode is deposited on the prepared silicon wafer using DC magnetron sputtering. Subsequently, using an impurity Hf target, Y-doped HfO_2_ thin films are grown under different parameter conditions via RF magnetron sputtering, and the surface morphology and crystal structure of the thin films are tested. Next, a TiN top electrode is grown using DC magnetron sputtering. The top electrode is deposited using a mask template to form a circular electrode with a diameter of 1 mm. After rapid thermal annealing at 700 °C, electrical performance tests are conducted.

TiN is widely used as an electrode material for ferroelectric HfO_2_-based devices due to its high electrical conductivity and good compatibility with HfO_2_-based thin films. In this study, TiN thin film electrodes were prepared using DC magnetron sputtering. The substrate used was a P-type (111) single crystal silicon wafer. Prior to the experiment, the substrate was placed in a solution of anhydrous ethanol and isopropanol for ultrasonic cleaning for 20 min. After drying with nitrogen gas, the target material and substrate were placed in the vacuum chamber. The vacuum system was activated, and the base vacuum was maintained at 2 × 10^−3^ Pa. The heating power supply was then activated to heat the substrate, and the target material was pre-sputtered to clean the surface contamination. A high-purity Ti target was used, with a purity of 99.99%, dimensions of 60 × 5 mm, a substrate material of P-Si (111), and a thickness of 1 mm. The Ar gas purity was 99.999%, N_2_ purity is 99.999%, working pressure is 0.5 Pa, DC power is 140 W, Ar:N_2_ ratio is 30:3, substrate temperature is 300 °C, and deposition time is 10 min to obtain a 120 nm TiN film.

RF magnetron sputtering was used to deposit Y-doped HfO_2_ thin films, with a base vacuum of 2.0 × 10^−3^ Pa, working pressure of 1.0 Pa, sputtering power of 200 W, Ar flow rate of 40 sccm, and O_2_ flow rate of 30 sccm. A main target material of Hf with a diameter of 60 nm, thickness of 3 nm, and purity of 99.99% was used. A small circular hole of 2 mm was fabricated within the sputtering zone of the Hf target, and Y plates with a diameter of 10 nm, thickness of 2 nm, and purity of 99.99% were uniformly embedded in the sputtering runway region. The doping concentration was controlled by adjusting the number of Y plates.

The film thickness is measured using the Newview-9000 non-contact 3D surface profiler was manufactured by ZYGO Corporation (Middlefield, CT, USA) with steps reserved during preparation. The film surface morphology is observed using The MultiMode 8 atomic force microscope (AFM) was manufactured by Bruker Corporation (Billerica, MA, USA). The molecular structure of the film is analyzed using a Raman spectrometer was manufactured by HORIBA Scientific (Longjumeau, Île-de-France, France). The crystal structure of the film was observed using The grazing incidence X-ray diffractometer (GIXRD, Bruker D8 model) was manufactured by Bruker Corporation (Billerica, MA, USA). The film devices were annealed using an RTP500 rapid annealing furnace was manufactured by Beijing Zhongke Tongzhi Technology Co., Ltd. (Beijing, China). The I-V curve test and P-E hysteresis loop test were conducted using the TF2000 ferroelectric tester was manufactured by aixACCT Systems GmbH (Erftstadt, Germany) to measure the leakage current and the maximum polarization intensity of the device structure.

A Thermo Scientific K-Alpha X-ray photoelectron spectrometer (XPS) was used for quantitative analysis of the elements in the thin film, the X-ray photoelectron spectrometer (XPS) was manufactured by Thermo Fisher Scientific (Waltham, MA, USA). Yielding concentrations of 4.71 mol.%, 6.44 mol.%, and 8.24 mol.% for 2, 3, and 4 Y plates, respectively. Previous studies on doping concentration revealed that the 4 Y plates doping exhibits superior electrical performance.

## 3. Results and Discussion

**Figure 2:**
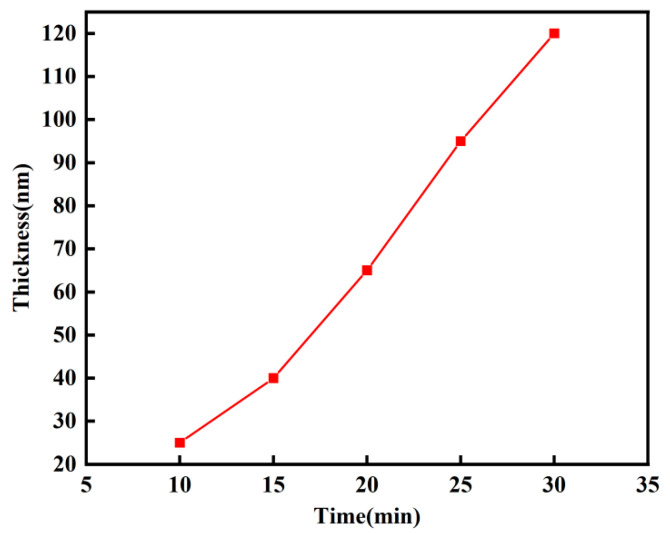
Thickness diagrams of Y-HfO_2_ films deposited at different times.

On the TiN thin film prepared by DC magnetron sputtering as the bottom electrode, Y-doped HfO_2_ thin films of different thicknesses were prepared using RF magnetron sputtering. The thickness was controlled by the deposition time, with deposition times of 10, 15, 20, 25, and 30 min selected for the experiment. The thicknesses of the resulting thin films are shown in Figure 2.

The film thicknesses are 25, 40, 65, 95, and 120 nm, showing a stepwise change. It was found that the film thickness is roughly linearly correlated with the deposition time. Under ideal conditions and stable deposition conditions, if the deposition rate remains constant, the thickness of the HfO_2_-based thin film is linearly related to the deposition time [14]. As the thickness of the HfO_2_ base film increases, the surface state of the film also changes, which can affect the subsequent deposition of atoms. Thinner films have relatively smooth surfaces, making it easier for atoms to adhere and deposit. However, once the film reaches a certain thickness, the surface may develop protrusions or defects, complicating the subsequent deposition process [15]. These surface features may hinder atomic diffusion and deposition, leading to reduced deposition rates and a deviation from the linear relationship between thickness and deposition time.

### 3.1. Surface Morphology of Y-HfO_2_ Thin Films of Different Thicknesses

**Figure 3:**
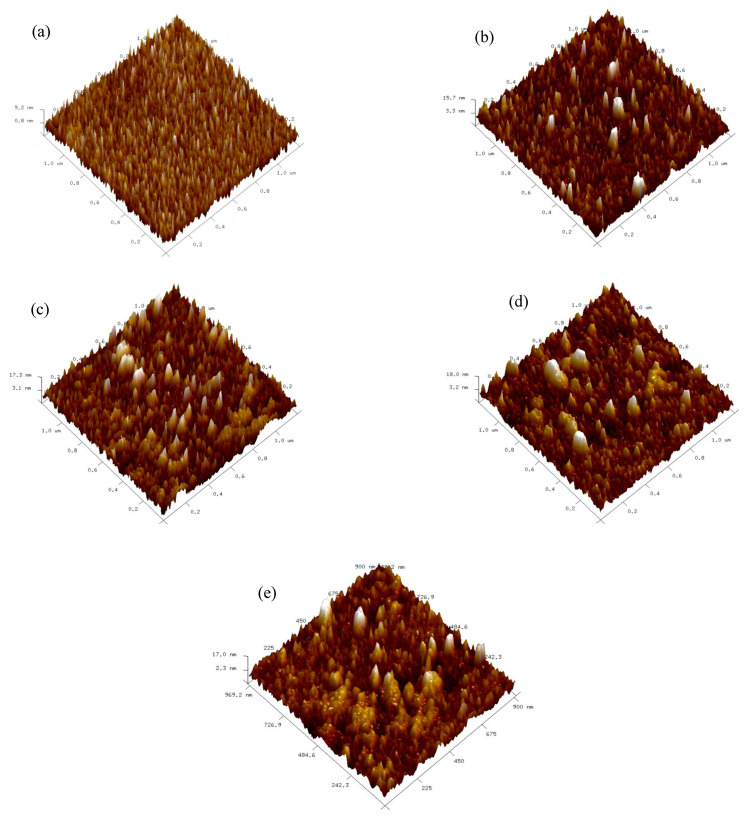
AFM test results of Y-HfO_2_ thin films at different thicknesses; (**a**): 25 nm; (**b**): 40 nm; (**c**): 65 nm; (**d**): 95 nm; (**e**): 120 nm.

All samples were first subjected to AFM testing to observe their surface morphology. Prior to testing, they were ultrasonically cleaned in anhydrous ethanol and dried with N_2_. The results are shown in Figure 3 below.

As the film thickness increases, the surface roughness (Rq) significantly increases, with values of 2.42 nm, 3.22 nm, 4.03 nm, 5.10 nm, and 6.05 nm from sample a to e, respectively. This phenomenon is primarily attributed to lattice distortion caused by the difference in atomic radii between Y and Hf atoms. When the film is thinner, the Y atom doping quantity is relatively low, resulting in limited lattice distortion that is localized, and the atomic deposition process remains relatively ordered, leading to a lower surface roughness of Rq = 2.42 nm. As the film thickness increases, the Y atom doping quantity rises, exacerbating lattice distortion and expanding its scope. This enhanced lattice distortion severely disrupts the ordered atomic deposition process, causing atoms to arrange in a more disordered manner on the surface, resulting in an increase in surface roughness Rq from 2.42 nm to 6.05 nm and a decrease in uniformity.

### 3.2. Crystal Structure of Y-HfO_2_ Thin Films of Different Thicknesses

**Figure 4:**
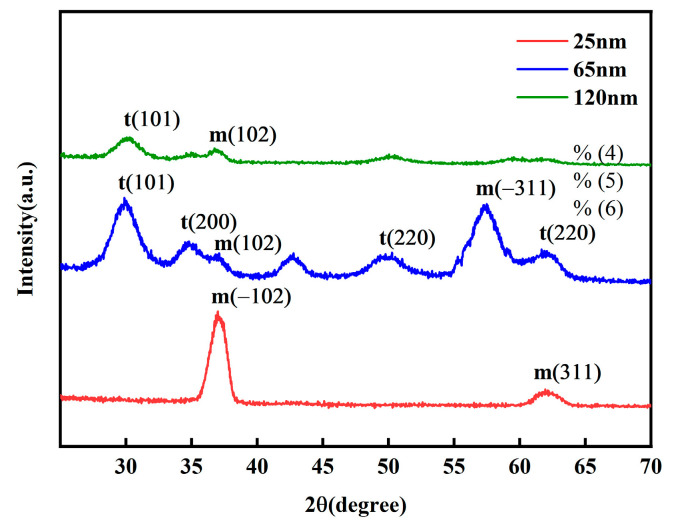
Grazing-incidence XRD patterns of Y-HfO_2_ thin films with different thicknesses.

The grazing incidence X-ray diffraction (XRD) results, as shown in Figure 4 reveal the evolution of the crystalline phase in the thin films with varying thickness. In the 25 nm film, the higher degree of freedom in surface atomic arrangement favors the formation of the energetically stable monoclinic phase (m). When the thickness increases to 65 nm, the crystalline structure transitions to a predominantly tetragonal phase (t), effectively suppressing the formation of the monoclinic phase (m). In the 120 nm thick film, high-temperature annealing promotes ordered lattice arrangement, further stabilizing the tetragonal phase (t) and increasing the grain size. This leads to a reduction in defect density; however, the enlarged grains are accompanied by decreased mobility of ferroelectric domain walls, which may result in the degradation of macroscopic ferroelectric properties.

Grain size calculations using the Scherrer equation based on the XRD patterns indicate that at a diffraction angle of 30.0°, both the 65 nm and 120 nm films exhibit a diffraction peak corresponding to the tetragonal t(101) phase, with grain sizes increasing from 3.5 nm to 6.27 nm. This is attributed to the longer deposition time required for thicker films, during which the substrate undergoes extended ion bombardment and thermal accumulation, promoting atomic diffusion and grain growth. At a diffraction angle of 36.9°, all samples show a diffraction peak associated with the monoclinic m(102) phase. The grain size of the 25 nm film is 6.96 nm, which can be ascribed to the higher atomic mobility facilitating the formation of larger nuclei. At 65 nm, the grain size decreases to 3.52 nm, likely due to the nucleation of a large number of new grains and competitive growth during the transition from a metastable phase to a stable phase. At 120 nm, the grain size significantly increases to 12.09 nm, owing to the longer deposition time providing an “annealing-like” effect that enhances atomic diffusion and grain coarsening.

**Figure 5:**
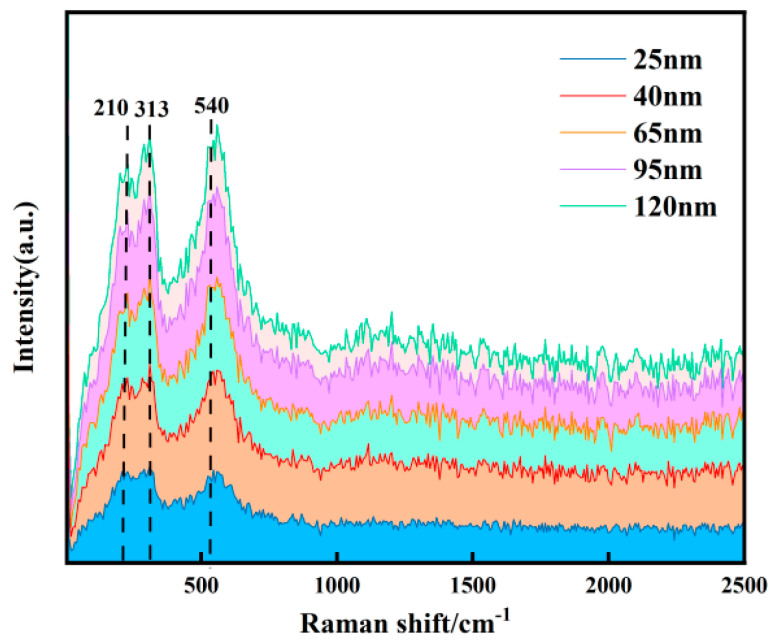
Raman test results for Y-HfO_2_ thin films of different thicknesses.

The Raman spectroscopy results are shown in Figure 5. All samples exhibit distinct characteristic peaks at 210 cm^−1^ (Y–O bond), 313 cm^−1^ (Hf–O bond), and 540 cm^−1^ (local vibration of Y^3+^), confirming the effectiveness of the Y-doped hafnium target. The positions of these characteristic peaks remain largely unchanged across different thicknesses, indicating that the fundamental crystal structure of the films remains unaltered. The intensity of all characteristic peaks increases significantly with increasing film thickness (from 25 nm to 120 nm). The enhancement of the 313 cm^−1^ peak is primarily attributed to the signal superposition effect resulting from the increased density of Hf–O bonds in thicker films. The enhancement of the 540 cm^−1^ peak may be due to the improved effect of Y doping. The variation in thickness is accompanied by an evolution in the internal structure of the films. The 25 nm film, likely due to instability during the initial growth stage, contains numerous defects, disordered regions, and non-uniformly distributed internal stresses concentrated at the surface and interface [16]. These factors can suppress atomic vibrations. As the thickness increases, the HfO_2_ crystal phase becomes more complete, and the long-range coordination of Hf–O bond vibrations is enhanced. Simultaneously, the distribution and role of Y atoms within the lattice become more stable [17]. Together, these contribute to an improvement in crystallinity, resulting in a stronger Raman signal and a systematic enhancement in the intensity of the characteristic peaks. The Hf–O bond vibration corresponding to the peak at 313 cm^−1^ is very close to the A_1_ g mode of the tetragonal phase. The presence of this peak in films of different thicknesses suggests that the 25 nm, 65 nm, and 120 nm samples may all possess the tetragonal phase [18].

### 3.3. Electrical Properties of Y-HfO_2_ Thin Films with Different Thicknesses

A TiN top electrode was prepared using direct current magnetron sputtering, resulting in a three-layer (TiN/Y-HfO_2_/TiN) thin film device, where the bottom TiN and top TiN electrodes each have a thickness of approximately 120 nm. After all samples were prepared, they were annealed in an RTP500 rapid annealing furnace using N_2_ at a heating rate of 35 °C/s to 700 °C and held for 40 s. Finally, the leakage current and hysteresis loop of the devices were measured using a ferroelectric tester to investigate the effect of film thickness on the electrical properties of the devices.

**Figure 6:**
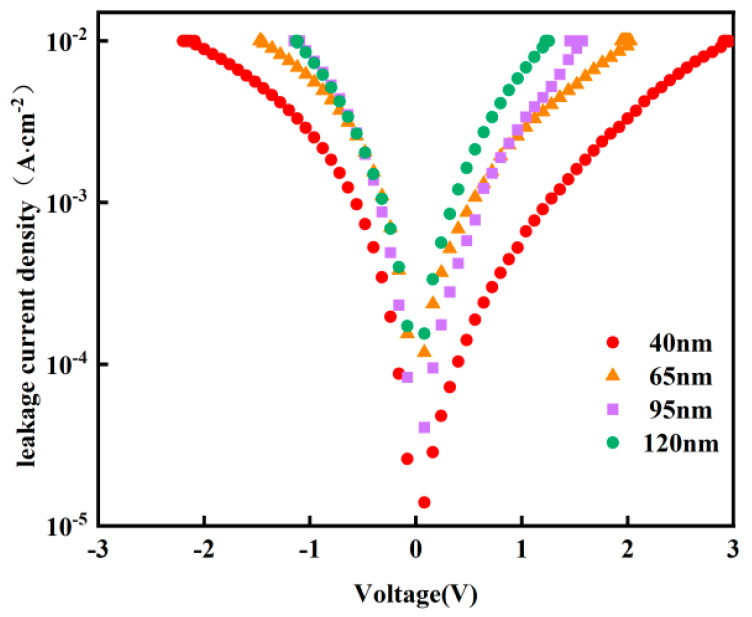
Leakage current test diagrams of TiN/Y-HfO_2_/TiN structures prepared from Y-HfO_2_films of different thicknesses.

Figure 6 shows the I-V characteristic curves of Y-HfO_2_ thin film devices with different thicknesses. The test range was from −2 V to 2 V. The leakage current density of all thicknesses increased with the increase in the absolute value of the applied voltage. At the same test voltage, the thickness of the thin film significantly affected the leakage current density, with the 40 nm thin film exhibiting the lowest leakage current density. As the thickness increases to 65 nm, 95 nm, and 120 nm, the leakage current density shows a noticeable upward trend. Near ±2 V, the 120 nm film exhibits the highest leakage current density. The 40 nm film has a shorter carrier transport path, thereby reducing the probability of tunneling and thermally excited conduction. It also has a lower surface defect density, reducing carrier recombination centers. The film exhibits better uniformity, resulting in a more uniform electric field distribution, which avoids local distortion and breakdown. The dielectric constant and breakdown field strength are closer to ideal conditions, effectively suppressing leakage current. The 120 nm film has uneven grain growth, leading to an accumulation of defect density and non-uniform internal electric field distribution. This makes it prone to local breakdown at defects or grain boundaries, causing a significant increase in leakage current.

**Figure 7:**
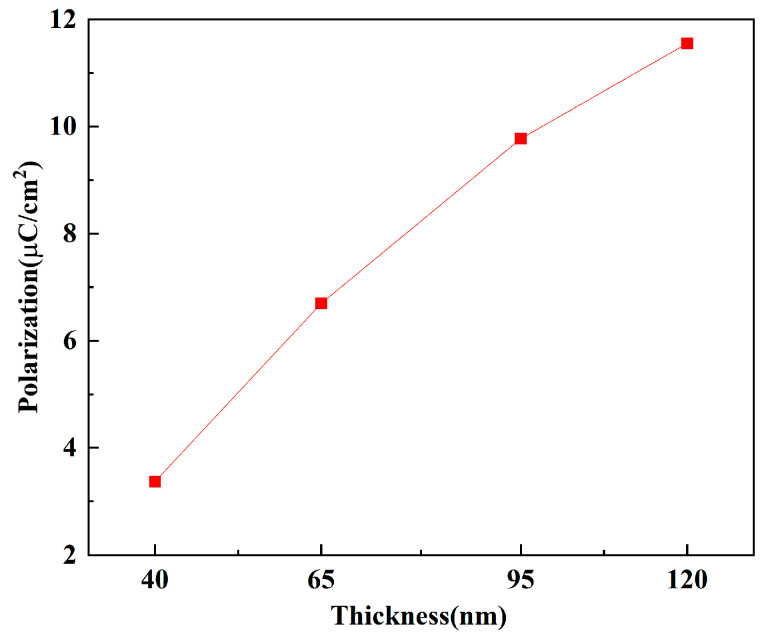
Diagram of the maximum polarization intensity of TiN/Y-HfO_2_/TiN structures prepared from Y-HfO_2_ films of different thicknesses.

Figure 7 shows the maximum polarization intensity of the TiN/Y-HfO_2_/TiN structure fabricated with different thicknesses of Y-HfO_2_ films. The polarization intensity of the films significantly increases with the thickness, from 3.36 μC/cm^2^ at 40 nm to 11.5 μC/cm^2^ at 120 nm. The external driving electric field required to achieve the same polarization intensity increases with the increase in film thickness. At 40 nm, the polarization intensity rapidly rises and approaches saturation under a lower electric field. The Y ion segregation and interface defect formation create a strong local electric field, effectively promoting the directional arrangement of electric dipoles. The edge effect of the film generates a high local electric field, significantly reducing the polarization threshold. For the 120 nm film, a higher electric field is required to drive the polarization to reach the saturation value. It mainly relies on the body phase defects to drive the polarization, and requires a higher external field to activate more polarization units. The larger domain size causes the domain wall movement to be affected by defect scattering, requiring a higher electric field to overcome the energy barrier, resulting in a slow increase in polarization.

## 4. Conclusions

(1) Y-HfO_2_ thin films of different thicknesses were prepared on TiN bottom electrodes using RF magnetron sputtering technology. Film thickness measurements showed an approximate linear relationship with deposition time. AFM surface morphology analysis indicated that the surface roughness of the films increased significantly with thickness, from 2.42 nm at 25 nm thickness to 6.05 nm at 120 nm thickness, with reduced uniformity. The 25 nm film exhibited a uniform and ordered surface, while the 120 nm film showed a loose surface with reduced order.

(2) Raman spectroscopy and XRD were employed to characterize the chemical bonding and crystalline phase structure of Y-HfO_2_ films with varying thicknesses. The XRD results indicate that the crystalline phase of the films transitions from monoclinic (m) to tetragonal (t) with increasing thickness. The grain size of the tetragonal phase (t) increases from 3.5 nm for the 65 nm-thick film to 6.27 nm for the 120 nm-thick film. Meanwhile, the grain size of the monoclinic phase (m) measures 6.96 nm for the 25 nm-thick film, decreases to 3.52 nm for the 65 nm-thick film, and then significantly increases to 12.09 nm for the 120 nm-thick film. The tetragonal phase (t) characteristic peaks are particularly prominent in the 65 nm-thick film. The Raman results show that vibration peaks corresponding to Y–O and Hf–O bonds are detected in films of all thicknesses. With increasing film thickness, the Raman peak intensities enhance, but no significant shift in the characteristic peak positions is observed.

(3) The TiN top electrode was fabricated by DC magnetron sputtering. Three-dimensional integration of Y-HfO_2_ films of different thicknesses was carried out to form a TiN/Y-HfO_2_/TiN structure device. Electrical performance tests were conducted after rapid thermal annealing at 700 °C. Leakage current tests indicated that as the thickness increased, the leakage current density also increased, mainly due to the increase in internal defects caused by the increase in the physical thickness of the films; the polarization strength increased with the increase in film thickness, and the maximum polarization strength of the 120 nm Y-HfO_2_ film reached 11.5 μC/cm^2^.

## Data Availability

The original contributions presented in this study are included in the article. Further inquiries can be directed to the corresponding author.
